# Long-Term Mortality and Health-Related Quality of Life After Continuous Versus Intermittent Renal Replacement Therapy in ICU Survivors: A Secondary Analysis of the Quality of Life After ICU Study

**DOI:** 10.1177/08850666231224392

**Published:** 2024-01-09

**Authors:** Mariana Martins Siqueira Santos, Daniel Sganzerla, Isabel Jesus Pereira, Regis Goulart Rosa, Cristina Granja, Cassiano Teixeira, Luís Azevedo

**Affiliations:** 1 MEDCIDS – Medicina da Comunidade, Informação e Decisão em Saúde, Department of Community Medicine, Information and Health Decision Sciences, Faculty of Medicine, 26706 University of Porto, Porto, Portugal; 2 CINTESIS@RISE – Centre for Health Technology and Services Research & Associate Laboratory – Health Research Network, 26706 University of Porto, Porto, Portugal; 3 Unimed, Porto Alegre, Brazil; 4 Polyvalent Intensive Care Medicine Service, Centro Hospitalar de Gaia/Espinho, Vila Nova de Gaia, Portugal; 5 Faculty of Medicine, 26706 University of Porto, Porto, Portugal; 6 CriticalMed – Critical Care & Emergency Medicine, 451731 CINTESIS – Center for Health Technology and Services Research, University of Porto, Porto, Portugal; 7 156417 Research Projects Office, Hospital Moinhos de Vento, Porto Alegre, Brazil; 8 Brazilian Research in Intensive Care Network (BRICNet), São Paulo, Brazil; 9 Research Unit, INOVA Medical, Porto Alegre, Brazil; 10 Intensive Care Department, Centro Hospitalar Universitário de São João, Porto, Portugal; 11 Anaesthesiology Department, 285211 Centro Hospitalar Universitário São João, Porto, Portugal; 12 Department of Surgery and Physiology, Faculdade de Medicina, University of Porto, Porto, Portugal; 13 Intensive Care Department, 37895 Hospital de Clínicas de Porto Alegre, Porto Alegre, Brazil; 14 Post-Graduation Program in Rehabilitation Sciences, Universidade Federalde Ciências da Saúde de Porto Alegre (UFCSPA), Porto Alegre, Brazil

**Keywords:** critical care, acute kidney injury, intermittent renal replacement therapy, continuous renal replacement therapy, late mortality, quality of life

## Abstract

**Purpose:** We assessed long-term outcomes in intensive care unit (ICU) survivors with acute kidney injury (AKI) submitted to intermittent or continuous renal replacement therapy (RRT) for comparisons between groups. **Methods:** The multicenter prospective cohort study included 195 adult ICU survivors with an ICU stay >72 h in 10 ICUs that had at least one episode of AKI treated with intermittent RRT (IRRT) or continuous RRT (CRRT) during ICU stay. The main outcomes were mortality and health-related quality of life (HRQoL). Hospital readmissions and physical dependence were also assessed. **Results:** Regarding RRT, 83 (42.6%) patients received IRRT and 112 (57.4%) received CRRT. Despite the similarity regarding sociodemographic characteristics, pre-ICU state of health and type of admission between groups, the risk of death (23.5% vs 42.7%; *P *< .001), the prevalence of sepsis (60.7%) and acute respiratory distress syndrome (17%) were higher at ICU admission among CRRT patients. The severity of critical illness was higher among CRRT patients, regarding the need for mechanical ventilation (75.0% vs 50.6%, *P* = .002) and vasopressors (91.1% vs 63.9%, *P* < .001). One year after ICU discharge, 67 of 195 ICU survivors died (34.4%) and, after adjustment for confounders, there were no significant differences in mortality when comparing IRRT and CRTT patients (34.9% vs 33.9%; *P = *.590), on HRQoL in both physical (41.9% vs 42.2%; *P *= .926) and mental dimensions (57.6% vs 56.6%; *P *= .340), and on the number of hospital readmissions and physical dependence. **Conclusions:** Our study suggests that among ICU survivors RRT modality (IRRT vs CRRT) in the ICU does not impact long-term outcomes after ICU discharge.

## Introduction

Acute kidney injury (AKI) is a common complication associated with critical illnesses, with both short and long-term unfavorable outcomes.^1,2^ AKI may occur in over 40% of patients in the intensive care unit (ICU) and is associated with adverse outcomes, such as increased length of stay, short- and long-term mortality, and end-stage renal disease.^3–6^ Up to 40% of ICU patients will need renal replacement therapy (RRT) due to AKI, and 13% to 21% of them will require RRT at hospital discharge.^7,8^ The ICU mortality rate can reach up to 65% for ICU AKI-RRT patients.^9^

The need for RRT is associated with an in-hospital mortality of about 50%.^2,10^ Currently, several RRT modalities may be used for AKI treatment in the ICU, and RRT could be intermittent or continuous, according to clinical recommendations.^11^ Regardless of the modality that is used, indications for the commencement of RRT for severe AKI patients are the same for all modalities. Intermittent renal replacement therapy (IRRT) is used for chronic kidney disease (CKD) patients receiving hemodialysis 3 times a week, 3 h to 5 h each session, with high flow rates to maintain the patient's hemodynamic stability. It removes solutes by diffusion and is more suited for patients requiring rapid substance removal. It is often less expensive and, if citrate is not used, requires less anticoagulation during RRT than continuous renal replacement therapy (CRRT)^12^ and can be used as an alternative in resource-limiting settings.^13^ IRRT may be associated with an increased risk of hypotension because of the removal of a large amount of fluid over a short time.^14^ CRRT provides slow and continuous kidney support. Compared with IRRT, CRRT tends to be associated with less cerebral edema because of a more physiological and slow removal of solutes,^15^ which can remove fluid steadily over a more extended time and is available 24 h a day to prevent fluid overload. It has been widely accepted that it should be preferentially used for critically ill patients with hemodynamic instability.^16^

However, long-term, treatment-modality, and disease-severity-unrelated mortality of up to 65% may be expected 2 years after ICU discharge for AKI-RRT patients.^17^ Despite the advances in RRT over the last few years, the immediate and long-term effects of different dialysis modalities used in the ICU are still a concern. Although the evidence is limited, some observational studies suggest that CRRT is associated with better kidney outcomes, mainly less need for chronic dialysis after hospital discharge.^18,19^ Nonetheless, prospective randomized studies comparing CRRT and IRRT could not find survival benefits.^14,20^ Considering the limited evidence available, this study aimed to compare intermittent and continuous RRT modalities regarding long-term mortality, health-related quality of life (HRQoL), hospital readmissions, and physical dependence 1 year after ICU discharge.

## Methods

### Study Design

We performed a secondary analysis of the Quality of Life After ICU study, a multicenter prospective cohort investigation that included patients discharged from 10 tertiary medical-surgical ICUs in Brazil (detailed in the electronic supplemental material [ESM]).^21^ All consecutive ICU survivors were recruited before hospital discharge (24 to 120 h after ICU discharge). They were followed up with structured telephone interviews performed by trained researchers 12 months after ICU discharge. The patients were recruited from May 2014 to December 2017 after approval by institutional review boards of all the participating centers. Consent for participation was obtained from all study participants or their proxies before hospital discharge.

### Participants

Patients aged ≥ 18 years who stayed at least for 72 h at the ICU for medical or surgical admissions and who had AKI and needed RRT (CRRT or IRRT) during the ICU stay were consecutively included after ICU discharge. Patients were excluded if they had severe preexisting CKD, according to the Charlson Comorbidity Index (CCI) criteria, and previous RRT, were transferred from another hospital's ICU to the study ICU, discharged directly from the ICU to home or to another hospital, when readmission to the ICU within 24 h of ICU discharge occurred and, for patients with communication difficulties, if proxies could not be reached. Refusal or withdrawal of agreement to participate, previous enrollment in the study, and unavailable telephone contact were also exclusion reasons. All patients alive at the time of ICU discharge who met the eligibility criteria were included in the long-term follow-up analysis. Patients receiving both CRRT and IRRT were considered for the CRRT group, as CRRT is usually applied in more severe cases presenting hemodynamic instability, according to the Kidney Disease Improving Global Outcomes (KDIGO) guidelines.^22,23^

### Outcomes

The primary outcome was the incidence of all-cause mortality one year after ICU discharge. Secondary outcomes were HRQoL, physical dependence, and new hospital admissions 12 months after ICU discharge. We eventually assessed some meaningful determinants of long-term outcomes of AKI treated with RRT patients: characteristics of acute critical illness and previous health status.

### Data Collection

Sociodemographic characteristics, pre-ICU health status, and characteristics of the critical illness were collected using structured face-to-face interviews performed at the moment of patient enrollment and through site investigators’ review of ICU charts.

Previous health statuses were assessed using the CCI^24^ dichotomized as low (score 0 or 1) or high comorbidity (≥ 2). The Barthel Index (BI) was used to assess physical functional statuses. This index classifies functional impairment into 3 categories based on specific cutoff values: severe impairment (0-50), moderate impairment (51-75), and mild to no impairment (76-100).^25^ We defined physical dependence as a BI less than or equal to 75. The severity of the critical illness was considered as the risk of death at ICU admission, defined as the predicted risk of hospital death according to the Acute Physiology and Chronic Health Evaluation II (APACHE-II)^26^ or the Simplified Acute Physiology Score 3 (SAPS 3), as some centers included in the study collected APACHE-II scores whereas other centers collected SAPS 3 scores.^27^ Sepsis and acute respiratory distress syndrome (ARDS) were defined according to the sepsis-II^28^ and Berlin definitions,^29^ respectively. Organ dysfunction was defined by the presence of any of the following conditions during the ICU stay: need of mechanical ventilation (MV), need of vasopressor, need of parenteral nutrition, need of blood or blood products transfusion, and delirium according to the Confusion Assessment Method for the ICU.^30^ Details about the RRT at the ICU and rehospitalization were assessed by site investigators using data in the medical charts. Health status at ICU discharge was evaluated in 24 to 120 h after ICU discharge, before hospital discharge. During the 12-month follow-up, telephone interviews were conducted by trained researchers not associated with patient care using structured telephone interviews after ICU discharge with a 30-day window period (15 days before and 15 days after the estimated date) to assess the impact of RRT on long-term outcomes. Patients were considered follow-up losses after 10 attempts of telephone contact without success. The outcomes analyzed during the 12-month follow-up period were the cumulative incidence of first unplanned readmission after hospital discharge, the number of readmissions per patient, and the mortality rates. HRQoL outcomes were assessed using the 12-Item Short Form Survey: Short Form 12 version 2 (SF-12v2); score range: 0 [worst] to 100 [best]) validated to telephone use. The SF-12v2 evaluates HRQoL through 8 domains (general health, physical functioning, physical role function, bodily pain, vitality, emotional role function, mental health, and social functioning) which are summarized in 2 dimensions: physical and mental.^31^

### Statistical Analysis

Data are expressed as median (interquartile range) for continuous variables and as counts and percentages for categorical variables. Comparisons between groups are presented as prevalence ratio and 95% confidence intervals (CI). Patients lost to follow-up were considered at risk until the date of last contact, at which point they were censored. Multivariate modeling was used to adjust primary outcome for baseline differences and acute critical illness (age, high comorbidity, sex, risk of death, sepsis or septic shock, and ARDS at ICU admission, need for MV and need for vasopressors at the ICU stay). Secondary outcomes were adjusted by multivariate modeling for age, sex, high comorbidity, and risk of death at ICU admission. The multivariate models included age, educational level, and comorbidities as categorical variables. We did not perform imputation for missing independent variables. Survival was graphically assessed using Kaplan-Meier curves. Mixed-effects Cox regressions were used to determine the association between independent variables and mortality. To account for the multicenter design's clustering effect, we considered the center (ICU) as a random effect. Results for mortality are presented as hazard ratios (HRs) and 95% CIs. *P*-values were two-sided, and statistical significance was set at .05. All analyses were performed using R version 3.5.1 (R Development Core Team).^32^

## Results

### Participants Characteristics

One hundred ninety-five patients without preexisting advanced CKD with the first need for RRT during ICU stay were included in the analysis. Characteristics of ICU AKI-RRT patients enrolled are summarized in Table 1. The median age was 67 years, and 55.5% were women. Median education attainment was 10 years, and the median monthly *per capita* household income was 538 U.S. dollars. Regarding pre-ICU health status, 64.6% of patients had a high CCI, and 2% were physically dependent. Previous history of depression and anxiety affected 20% and 19% of patients, respectively, and dementia was previously diagnosed in nearly 4%. Concerning critical illness, 24.6% were admitted to the ICU after surgery; at ICU admission, the median risk of death was 35.5%, 54.4% of participants were admitted with sepsis, and 12.3% presented ARDS.

**Table 1.** Baseline and Acute Critical Illness Characteristics of Participants in the Study.

	No. /Total (%)			
	Intermittent (*N* = 83)	Continuous (*N* = 112)	Prevalence ratio (PR)^a^ (95% CI)	*P*-value
**Sociodemographic characteristics**				
Age (years), median (IQR)	67.0 (48.5-79)	67.5 (57-76)	1 (0.99-1.01)	.759
Age ≥65 years	46/83 (55.4)	62/112 (55.4)	1 (0.78-1.27)	.993
Female sex	36/83 (43.4)	47/112 (42)	0.98 (0.76-1.25)	.844
Educational attainment (years), median (IQR)	8 (4-11)	11 (5-16)	1.03 (1.01-1.05)	.012
Higher education^b^	13/82 (15.9)	31/112 (27.7)	0.77 (0.6-0.98)	.031
Monthly “per capita” household income^c^, U.S. dollar—median (IQR)	444 (247-1115.7)	636 (405.4-1736.5)	1 (1-1)	.473
**Health status before admission to the ICU**				
Charlson comorbidity index—median (IQR)	2 (1-4.5)	2 (1-3)	0.98 (0.92-1.03)	.409
High comorbidity^d^	55/83 (66.3)	71/112 (63.4)	0.95 (0.74-1.22)	.675
Previous history of myocardial infarction	10/83 (12)	18/112 (16.1)	1.14 (0.84-1.55)	.396
Previous history of congestive heart failure	13/83 (15.7)	19/112 (17)	1.04 (0.76-1.43)	.805
Previous history of cerebrovascular disease	10/83 (12)	16/112 (14.3)	1.08 (0.78-1.51)	.636
Previous history of diabetes with chronic complications	10/83 (12)	11/112 (9.8)	0.9 (0.59-1.38)	.637
Previous history of mild chronic kidney disease^e^	9/83 (10.8)	5/112 (4.5)	0.6 (0.3-1.23)	.166
Previous history of dementia	3/83 (3.6)	4/112 (3.6)	0.99 (0.52-1.91)	.987
Previous history of depression	19/81 (23.5)	19/111 (17.1)	0.84 (0.59-1.18)	.310
Previous history of anxiety	17/81 (21)	20/112 (17.9)	0.92 (0.66-1.27)	.599
Barthel Index, median (IQR)	95 (85-100)	95 (85-100)	1 (1-1.01)	.242
Totally functional dependence	2/82 (2.4)	2/112 (1.8)		
Independent	38/82 (46.3)	55/112 (49.1)		
**Characteristics of acute critical illness**				
ICU Admission type			1.01 (0.85-1.2)	.919
Medical	64/83 (77.1)	83/112 (74.1)		
Surgical, elective	7/83 (8.4)	15/112 (13.4)		
Surgical, emergency	12/83 (14.5)	14/112 (12.5)		
Risk of death at ICU admission^f^, median (IQR)	23.5 (16.5-45.1)	42.7 (23.5-70.9)	1.01 (1-1.01)	<.001
Sepsis or septic shock^g^ at ICU admission	38/83 (45.8)	68/112 (60.7)	1.3 (1.01-1.67)	.044
Acute respiratory distress syndrome^h^ at ICU admission	5/83 (6)	19/112 (17)	1.46 (1.14-1.86)	.003
**Organ dysfunction during ICU stay**				
Need for mechanical ventilation	42/83 (50.6)	84/112 (75)	1.64 (1.20-2.24)	.002
Days of mechanical ventilation, median (IQR)	1 (0-8.5)	5 (0.8-10)	1.00 (1.00-1.01)	.376
Need for vasopressor	53/83 (63.9)	102/112 (91.1)	2.63 (1.52-4.56)	<.001
Need for parenteral nutrition	8/83 (9.6)	9/112 (8)	0.91 (0.57-1.46)	.708
Need for blood or blood products transfusion	28/83 (33.7)	41/112 (36.6)	1.05 (0.82-1.35)	.675
Delirium	28/83 (33.7)	50/112 (44.6)	1.21 (0.95-1.53)	.117
ICU-acquired infections^i^	24/83 (28.9)	35/112 (31.2)	1.05 (0.81-1.36)	.722
ICU length of stay, days, median (IQR)	9 (5-15.5)	11 (7-19.2)	1 (0.99-1.01)	.671
Hospital length of stay, days, median (IQR)	36 (20.5-66.5)	37 (25.8-60)	1 (1-1)	.936
**State of health immediately after ICU discharge (24 to 120 h)**				
Respondents—MMSE	48/83 (57.8)	64/112 (57.1)	0.99 (0.77-1.26)	.923
MMSE score, median (IQR)	24 (20-26)	23.5 (19-26)	0.99 (0.97-1.02)	.719
Cognitive dysfunction^j^	21/48 (43.8)	33/64 (51.6)	1.14 (0.83-1.58)	.413
Respondents—MRC	49/83 (59)	70/112 (62.5)	1.06 (0.83-1.37)	.627
MRC, median (IQR)	48 (44-60)	48 (42.2-56)	0.99 (0.98-1.01)	.455
Muscular weakness (MCR <48)	20/49 (40.8)	26/70 (37.1)	0.94 (0.68-1.28)	.689

Abbreviations: ICU, intensive care unit; IQR, interquartile range (p25; p75); MMSE, Mini-Mental State Examination; MRC, Medical Research Council scale.

^a^ Prevalence ratio (PR) ratio was used to compare groups.

^b^ Individuals holding a university degree.

^c^ Using the purchasing power parity conversion (Brazilian real to U.S. dollar). Purchasing power parities are the rates of currency conversion that equalize the purchasing power of different currencies by eliminating the differences in price levels between countries https://data.oecd.org/conversion/purchasing-power-parities-ppp.htm.

^d^ Charlson comorbidity index ≥2.

^e^ Defined according to CCI criteria.

^f^ The risk of death was calculated using established prediction equations for hospital death according to the Acute Physiology and Chronic Health Evaluation II score (APACHE-II) or the Simplified Acute Physiology Score-3 (SAPS 3).

^g^ According to the sepsis-II criteria.

^h^ According to Berlin criteria.

^i^ Pneumonia, bloodstream infection, or urinary tract infection according to the European Centre for Disease Prevention and Control criteria.

^j^ Mini Mental State Examination ≤21 if 4 years or less of educational attainment, or ≤24 if >4 years of educational attainment.

During the ICU stay, 64.6% of the patients were on MV, the median MV length was 4 days (0-10), 79.5% were on vasopressor support, and 35.4% needed blood or blood products transfusion. Delirium was diagnosed in 40% of ICU AKI-RRT patients, and 30.3% of the patients contracted a nosocomial infection. The median ICU length of stay was 9 (6-19) days, and the median in-hospital length was 37 (22-61) days.

Comparing groups, 83 (42.6%) patients received IRRT and 112 (57.4%) received CRRT. There were no significant differences in mean age and sex distribution and previous physical functional status, as assessed by BI. However, CRRT patients had higher education levels (15.9% vs 27.7%; *P *= .031).

Regarding critical illness, 77% of patients receiving IRRT and 74% of those treated with CRRT were admitted to the ICU due to medical reasons (*P *= .919). Although no differences were observed regarding the type of admission, patients treated with CRRT presented significantly worse disease severity at ICU admission.

The median risk of death at ICU admission was higher among CRRT patients (23.5% vs 42.7%; *P *< .001), and sepsis and ARDS were more often present at ICU admission among CRRT patients (60.7% vs 45.8%, *P *= .044; 17% vs 6%, *P *= .003). Significantly higher severity of critical illness among CRRT patients was also confirmed by the differences observed between groups regarding the need for MV and vasopressors (75.0% vs 50.6%, *P* = .002; 91.1% vs 63.9%, *P* < .001) and there were no significant differences between groups in cognition and physical functional status after the ICU stay.

### Long-Term Post-ICU Mortality

**Figure 1.**
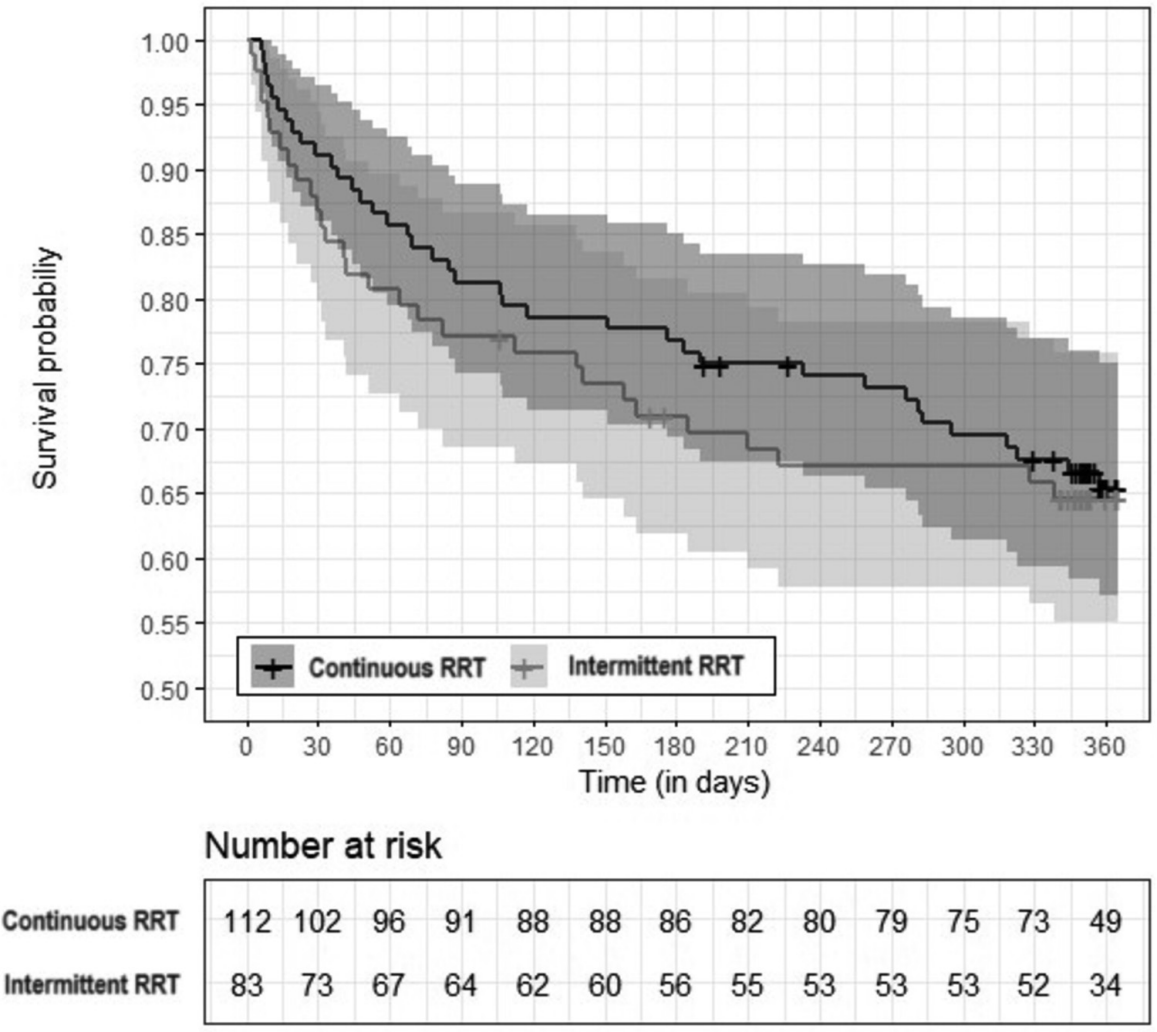
Survival data. Kaplan-Meier survival curve during the 12-month follow-up showing the comparison between CRRT versus IRRT groups (*P* = .999). The shaded areas of the Kaplan-Meier curve represent the 95% CI for both groups.

Abbreviation: CRRT, continuous renal replacement therapy; IRRT, intermittent renal replacement therapy.

During the 12-month follow-up, 67 of 195 ICU survivors died (34.4%). Figure 1 shows survival data over time for both groups. There were no significant differences in mortality one year after ICU discharge when comparing IRRT patients (34.9%) and CRRT patients (33.9%; *P = *.483). All baseline variables were included in the univariable analysis, and age (HR, 1.02; 95% CI [1.00-1.03]; *P* = .010) and pre-ICU high comorbidity (HR, 2.51; 95%CI, 1.45-4.36; *P* = .001) were the only variables associated with late mortality. Results of univariable analysis of the factors associated with RRT and late mortality are shown in Supplemental Table 1 (ESM).

In the multivariable analysis of variables associated with RRT modality and the primary outcome, pre-ICU high comorbidity (HR, 2.73; 95% CI [1.45-5.13]; *P* = .002) was the only variable independently associated. Site, RRT modality, sex, age, state of health before admission to the ICU (risk of death at ICU admission and previous ICU comorbidities), and relevant characteristics of acute critical illness (sepsis, ARDS, need for MV, and need for vasopressors), were also considered at the final adjusted model, due to their clinical relevance and according to previous literature. The results of the multivariable analysis are shown in Table 2. No significant differences were observed between IRRT and CRRT regarding long-term mortality (HR = 1.16, 95% CI [0.68-1.98], *P* = .590).

**Table 2.** Multivariable Analysis of Factors Associated With RRT Modality and Late Mortality.

	Hazard ratio^a^	95% CI	*P*-value
RRT modality (CRRT)	1.16	0.68-1.98	.590
Sociodemographic characteristics			
Age >65 years	1.55	0.90-2.66	.110
Female sex	1.04	0.63-1.70	.880
State of health before admission to the ICU			
High comorbidity^c^	2.73	1.45-5.13	.002
Characteristics of acute critical illness			
Risk of death at ICU admission^c^	1.01	1.00-1.02	.140
Sepsis or septic shock at ICU^d^	0.84	0.47-1.48	.540
ARDS^e^ at ICU	1.12	0.48-2.60	.790
Organ dysfunction during ICU stay			
Need of mechanical ventilation	0.77	0.41-1.44	.410
Need of vasopressor	1.20	0.60-2.40	.610

Abbreviations: RRT, renal replacement therapy; CRRT, continuous renal replacement therapy; ARDS, acute respiratory distress syndrome; CI, confidence interval; ICU, intensive care unit.

^a^ Adjusted by site.

^b^ Charlson comorbidity index >2.

^c^ The risk of death was calculated using established prediction equations for hospital death according to the Acute Physiology and Chronic Health Evaluation II score (APACHE-II) or the Simplified Acute Physiology Score-3 (SAPS 3).

^d^ According to the sepsis-II criteria.

^e^ According to Berlin criteria.

### Health-Related Quality of Life

Regarding long-term HRQoL, our data supports that those ICU survivors undergoing IRRT at the ICU had no significant differences in both physical (41.9 vs 42.2; *P *= .844) and mental dimensions (57.6 vs 56.6; *P *= .290) compared with those receiving CRRT. One year after ICU discharge, 43 patients responded to SF-12v2.

We also did not observe significant differences in the number of hospital readmissions and physical dependence at 12 months after ICU discharge (53.9% vs 65.7%; *P *= .339 and 22.2% vs 26.7%; *P *= .324, respectively) (Table 3).

**Table 3.** Study outcomes.

		Renal replacement therapy			
Outcomes in 12 months after ICU discharge	Total	Intermittent	Continuous	*P*-value	*Adjusted P*-value
Mortality rate—no./total no. (%)	67/195 (34.4)	29/83 (34.9)	38/112 (33.9)	.710	.483
SF12 Score^a^—median (IQR)					
** **Mental	57.2 (49.3-61.9)	57.6 (49.3-62.3)	56.6 (49.3-61)	.340	.290
** **Physical	41.9 (32.3-49.7)	41.9 (33-47.9)	42.2 (32.2-50.4)	.926	.844
Hospital Readmissions^b^—no./total no. (%)	112/184 (60.9)	41/76 (53.9)	71/108 (65.7)	.144	.339
Physical Dependence^c^—no./total no. (%)	26/105 (24.8)	10/45 (22.2)	16/60 (26.7)	.769	.324

^a^ Health-related Quality of Life (HRQoL).

^b^ Hospital readmissons in 12 months after ICU discharge.

^b^ Barthel index ≤75.

## Discussion

We conducted a 1-year follow-up analysis of 195 ICU AKI-RRT survivors after ICU discharge. We only included ICU survivors without a previous history of RRT or advanced CKD. Our study presents the following findings: first, late mortality until 12 months after ICU discharge was 34%. We believe this is one of the very few studies available on AKI-RRT ICU survivors presenting data on long-term (1-year) mortality. Recently, a population-based cohort study found a 1-year mortality rate after ICU admission up to 30% among general ICU survivors.^33^ We observed a homogeneous population in terms of sociodemographic characteristics and health status before admission to the ICU. No differences were found between the IRRT (*n* = 83) group and the CRRT (*n* = 112) group in terms of general characteristics (age and sex) and previous comorbidities (*P* = .759, *P* = .844, *P* = .409, respectively). However, CRRT patients had a higher risk of death and presented a higher prevalence of sepsis and ARDS at ICU admission. Despite that, considering the adjusted model, no significant differences were found between CRRT and IRRT mortality one year after ICU discharge.

Previous observational studies described that, among ICU survivors, initial treatment with IRRT might be associated with higher rates of dialysis dependence than CRRT.^20^ Still, results obtained in randomized trials showed no effect of CRRT improving renal recovery compared to IRRT.^34^ The results of our present cohort study are not consistent with some previous observational studies, as we have not found a significant association between the modality of RRT and improvement in renal recovery.^35^ However, limited information exists regarding the potential long-term effects on clinical outcomes beyond 3 months following ICU discharge.^36^ In this regard, the present cohort study is relevant because it adds a longer 1-year follow-up and does not confirm the effect of RRT.

Several RCTs and observational studies have explored the potential short-term (ICU or in-hospital) benefits of CRRT versus other RRT types. Their results have been synthesized in several systematic reviews and meta-analyses.^37–39^ In general, RCTs show no advantage of CRRT in mortality rates.^37,40^ CRRT did not demonstrate a significant advantage concerning other outcomes, such as the recovery of kidney function or length of stay in the ICU.^38^ However, the evidence on long-term survival and other long-term outcomes is scarce.^23^ In a single-center observational study with 1292 ICU AKI-RRT patients, the use of CRRT was associated with increased mortality after 1 year.^35^ In another study, an RCT with a 60-day survival as a primary endpoint, the mortality did not differ between the groups (IRRT vs CRRT).^14^ The relevance of the present study is to add to the very limited evidence regarding the potential long-term benefits of CRRT or the lack thereof. In 2016, Liang et al conducted an observational study that also concluded no significant differences in mortality 90 days after ICU discharge.^41^ In a recent metaanalysis, observational studies suggested a higher rate of dialysis dependence among survivors who initially received IRRT as compared with CRRT. However, RCTs showed no significant differences. Findings that primarily rely on data from observational trials may be susceptible to allocation and selection bias, not providing high-quality evidence to support the conclusion that CRRT leads to improved renal recovery.^20^ Regarding RCTs, studies provided no evidence for a long-term survival benefit of continuous versus intermittent RRT in ICU survivors.^14,34^ In our study, similarly to recent RCTs, the modality of RRT, either CRRT or IRRT, had no impact on mortality 1 year after ICU discharge.^36^ However, a prior cohort study presented reduced long-term survival rates after AKI-RRT, associated with the RRT modality selected at the onset of treatment, with higher mortality in the CRRT group.^35^ The inverted pattern observed in our study, compared to other observational studies, further reinforces the lack of high-quality data supporting that CRRT results in improved renal recovery than IRRT.^23^

Some cohort studies have reported an association between RRT at the ICU and long-term outcomes, namely, severe CKD and mortality for ICU survivors.^42–44^ The mortality rate among AKI-RRT patients is very high. A patient's death may be predicted by the causes of AKI (sepsis, cardiac surgery), clinical course (oliguria, pulmonary edema, hypotension, acidosis, lesion of other organs), and the need for CRRT,^45^ ie, if a patient survives to the acute episode while they are in the ICU, then the RRT modality will not influence its long-term mortality.

Our second finding was that there were no significant differences between CRRT and IRRT concerning hospital readmissions, HRQoL, and physical dependence. Vrettou et al recently published a Greek observational study supporting that preexisting comorbidities were one of the main factors related to worse HRQoL in critically ill patients 1 year after ICU discharge.^46^ Nearly all ICU patients leave the hospital with significant impairments in physical function, cognitive status, or both.^47^ Most of them require institutional care, and the incidence of hospital readmissions among general ICU patients during the first year after hospital discharge exceeds 40%.^48,49^

Our findings on long-term HRQoL align with those reported in previous studies, suggesting that the long-term HRQoL impairments following critical illness may stem from the physical function limitations, cognitive status impairments, and constraints associated with the AKI and RRT itself, rather than being attributed to the specific modality of RRT. Studies addressing long-term critically ill AKI-RRT survivors showed that HRQoL was comparable to non-AKI-RRT survivors but lower than in the general population.^50,51^ At a recent retrospective cohort study, AKI-RRT ICU survivors self-reported lower HRQOL, but there were no significant differences in cognitive function or emotional health compared to ICU survivors not receiving RRT.^52^

The severity of illness for ICU-AKI-RRT survivors is higher compared to non-AKI-RRT patients, and one-quarter of AKI-RRT patients presented long-term persistent dialysis dependency.^53,54^ As for mortality, hospital readmissions, HRQoL, and physical dependence may not be influenced by the RRT modality when the patient survives the acute episode.

The strengths of this study include: first, despite differences in the previous health status, mainly the number of comorbidities before ICU admission, the characteristics of acute critical illness during ICU treatment are similar for patients included in both groups. Second, patients were followed until 12 months after ICU discharge, and data concerning their HRQoL, physical dependence, and hospital readmissions were obtained in both groups of RRT modalities. Most previous studies did not have such a long follow-up period. Additionally, the multicenter prospective design may have promoted the standardization of eligibility criteria and prevented missing values, and the use of a starting point after discharge from the ICU for follow-up reinforces the perspective of post-ICU care.

Some limitations must be considered: First, despite being a multicentered study, our sample was limited to one middle-income country, and distinct sociocultural contexts may impact the RRT facilities available to participants after hospital discharge. Second, patients with short ICU stays (<72 h) and those who were discharged directly from the ICU to home or to another hospital were not included, which may impair the generalizability of results. Third, included patients were submitted to RRT at least once in the ICU. However, we did not assess whether patients were still on RRT at discharge. Fourth, the initial RRT modality upon ICU admission was not assessed as it was not a primary variable in the original study protocol.^55^ Likewise, the decision to include patients treated with both IRRT and CRRT within the CRRT group is a potential limitation. The long-term effects of treatment with both IRRT and CRRT may be more similar to treatment with only IRRT than to treatment with only CRRT. Fifth, our study is a real-world observational study, and participating ICUs exhibited some heterogeneity in their utilization of SAPS-3 and APACHE-2 at ICU admission. To address this, we decided to use the risk of death upon admission to the ICU as a proxy for the severity of acute critical illness. This approach allows us to account for the varying practices across ICUs while still assessing the overall risk.^56^^57^ Finally, we lacked sufficient data to determine the AKI stage when RRT was initiated, as neither the KDIGO criteria nor other relevant criteria were documented. Additionally, despite its limitations, we employed the CCI as the definition of CKD, as it offers a highly crude assessment of CKD status. We also did not control new RRT needs, and treatment limitations imposed after ICU discharge and propensity score-matched analysis was not performed to compensate for indication bias, as the number of events was too small.

## Conclusion

We found no evidence of differences in mortality, hospital readmissions, HRQoL, and physical dependence associated with the CRRT or IRRT among ICU AKI-RRT survivors 12 months after ICU discharge.

## Supplemental Material

sj-docx-1-jic-10.1177_08850666231224392 - Supplemental material for Long-Term Mortality and Health-Related Quality of Life After Continuous Versus Intermittent Renal Replacement Therapy in ICU Survivors: A Secondary Analysis of the Quality of Life After ICU Study Supplemental material, sj-docx-1-jic-10.1177_08850666231224392 for Long-Term Mortality and Health-Related Quality of Life After Continuous Versus Intermittent Renal Replacement Therapy in ICU Survivors: A Secondary Analysis of the Quality of Life After ICU Study by Mariana Martins Siqueira Santos, Daniel Sganzerla, Isabel Jesus Pereira, Regis Goulart Rosa, Cristina Granja, Cassiano Teixeira and Luís Azevedo in Journal of Intensive Care Medicine

## Abbreviations

AKI: acute kidney injury
APACHE-II: Acute Physiology and Chronic Health Evaluation II
ARDS: acute respiratory distress syndrome
BI: Barthel Index
CAM-ICU: Confusion Assessment Method for the ICU
CCI: Charlson Comorbidity Index
CI: confidence interval
CKD: chronic kidney disease
CRRT: continuous renal replacement therapy
ESM: electronic supplemental material
ESRD: end-stage renal disease
HR: hazard ratio
HRQoL: health-related quality of life
ICU: intensive care unit
IQR: interquartile range
IRRT: intermittent renal replacement therapy
MV: mechanical ventilation
PR: prevalence ratio
RRT: renal replacement therapy
SAPS-3: Simplified Acute Physiology Score 3
SF-12v2: Short Form 12 version 2

## References

[1] Nisula S Kaukonen KM Vaara ST , et al. Incidence, risk factors and 90-day mortality of patients with acute kidney injury in Finnish intensive care units: The FINNAKI study. Intensive Care Med. 2013;39(3):420-428. 10.1007/s00134-012-2796-5 23291734

[2] Morgera S Kraft AK Siebert G Luft FC Neumayer HH . Long-term outcomes in acute renal failure patients treated with continuous renal replacement therapies. Am J Kidney Dis. 2002;40(2):275-279. 10.1053/ajkd.2002.34505 12148099

[3] Hoste EA Bagshaw SM Bellomo R , et al. Epidemiology of acute kidney injury in critically ill patients: The multinational AKI-EPI study. Intensive Care Med. 2015;41(8):1411-1423. 10.1007/s00134-015-3934-7 26162677

[4] Hashemian SM Jamaati H Farzanegan Bidgoli B , et al. Outcome of acute kidney injury in critical care unit, based on AKI network. Tanaffos. 2016;15(2):89-95. 27904540 PMC5127620

[5] Libório AB Leite TT Neves FM Teles F Bezerra CT . AKI Complications in critically ill patients: Association with mortality rates and RRT. Clin J Am Soc Nephrol. 2015;10(1):21-28. 10.2215/cjn.04750514 25376761 PMC4284413

[6] Kellum JA Angus DC . Patients are dying of acute renal failure. Crit Care Med. 2002;30(9):2156-2157. 10.1097/00003246-200209000-00041 12352064

[7] Fagugli RM Patera F Battistoni S Mattozzi F Tripepi G . Six-year single-center survey on AKI requiring renal replacement therapy: Epidemiology and health care organization aspects. J Nephrol. 2015;28(3):339-349. 10.1007/s40620-014-0114-8 24935754

[8] Uchino S Kellum JA Bellomo R , et al. Acute renal failure in critically ill patients: A multinational, multicenter study. Jama. 2005;294(7):813-818. 10.1001/jama.294.7.813 16106006

[9] Susantitaphong P Cruz DN Cerda J , et al. World incidence of AKI: A meta-analysis. Clin J Am Soc Nephrol. 2013;8(9):1482-1493. 10.2215/cjn.00710113 23744003 PMC3805065

[10] Ishani A Xue JL Himmelfarb J , et al. Acute kidney injury increases risk of ESRD among elderly. J Am Soc Nephrol. 2009;20(1):223-228. 10.1681/asn.2007080837 19020007 PMC2615732

[11] Mercado MG Smith DK Guard EL . Acute kidney injury: Diagnosis and management. Am Fam Physician. 2019;100(11):687-694. 31790176

[12] Manns B Doig CJ Lee H , et al. Cost of acute renal failure requiring dialysis in the intensive care unit: Clinical and resource implications of renal recovery. Crit Care Med. 2003;31(2):449-455. 10.1097/01.Ccm.0000045182.90302.B3 12576950

[13] Sankarasubbaiyan S Janardan JD Kaur P . Outcomes and characteristics of intermittent hemodialysis for acute kidney injury in an intensive care unit. Indian J Nephrol. 2013;23(1):30-33. 10.4103/0971-4065.107193 23580802 PMC3621235

[14] Vinsonneau C Camus C Combes A , et al. Continuous venovenous haemodiafiltration versus intermittent haemodialysis for acute renal failure in patients with multiple-organ dysfunction syndrome: A multicentre randomised trial. Lancet. 2006;368(9533):379-385. 10.1016/s0140-6736(06)69111-3 16876666

[15] Ronco C Bellomo R Brendolan A Pinna V La Greca G . Brain density changes during renal replacement in critically ill patients with acute renal failure. Continuous hemofiltration versus intermittent hemodialysis. J Nephrol. 1999;12(3):173-178. 10440514

[16] Kes P Ljutić D Basić-Jukić N Brunetta B . Indications for continuous renal function replacement therapy. Acta Med Croatica. 2003;57(1):71-75. (Indikacije za kontinuirano nadomjestanje bubrezne funkcije.). 12876869

[17] Van Berendoncks AM Elseviers MM Lins RL . Outcome of acute kidney injury with different treatment options: Long-term follow-up. Clin J Am Soc Nephrol. 2010;5(10):1755-1762. 10.2215/cjn.00770110 20634328 PMC2974373

[18] Bell M Granath F Schön S Ekbom A Martling CR . Continuous renal replacement therapy is associated with less chronic renal failure than intermittent haemodialysis after acute renal failure. Intensive Care Med. 2007;33(5):773-780. 10.1007/s00134-007-0590-6 17364165

[19] Uchino S Bellomo R Kellum JA , et al. Patient and kidney survival by dialysis modality in critically ill patients with acute kidney injury. Int J Artif Organs. 2007;30(4):281-292. 10.1177/039139880703000402 17520564

[20] Schneider AG Bellomo R Bagshaw SM , et al. Choice of renal replacement therapy modality and dialysis dependence after acute kidney injury: A systematic review and meta-analysis. Intensive Care Med. 2013;39(6):987-997. 10.1007/s00134-013-2864-5 23443311

[21] Rosa RG Falavigna M Robinson CC , et al. Early and late mortality following discharge from the ICU: A multicenter prospective cohort study. Crit Care Med. 2020;48(1):64-72. 10.1097/ccm.0000000000004024 31609775

[22] KDIGO Board members. Kidney Int Suppl (2011). 2012;2(1):3. 10.1038/kisup.2012.3 25028632 PMC4089615

[23] Wang AY Bellomo R . Renal replacement therapy in the ICU: Intermittent hemodialysis, sustained low-efficiency dialysis or continuous renal replacement therapy? Curr Opin Crit Care. 2018;24(6):437-442. 10.1097/mcc.0000000000000541 30247213

[24] Charlson ME Pompei P Ales KL MacKenzie CR . A new method of classifying prognostic comorbidity in longitudinal studies: Development and validation. J Chronic Dis. 1987;40(5):373-383. 10.1016/0021-9681(87)90171-8 3558716

[25] Mahoney FI Barthel DW . Functional evaluation: The Barthel Index. Md State Med J. 1965;14(1):61-65. 14258950

[26] Knaus WA Draper EA Wagner DP Zimmerman JE . APACHE II: A severity of disease classification system. Crit Care Med. 1985;13(10):818-829. 3928249

[27] Le Gall JR Lemeshow S Saulnier F . A new simplified acute physiology score (SAPS II) based on a European/north American multicenter study. Jama. 1993;270(24):2957-2963. 10.1001/jama.270.24.2957 8254858

[28] Dellinger RP Levy MM Rhodes A , et al. Surviving sepsis campaign: International guidelines for management of severe sepsis and septic shock: 2012. Crit Care Med. 2013;41(2):580-637. 10.1097/CCM.0b013e31827e83af 23353941

[29] Ranieri VM Rubenfeld GD Thompson BT , et al. Acute respiratory distress syndrome: The Berlin definition. Jama. 2012;307(23):2526-2533. 10.1001/jama.2012.5669 22797452

[30] Ely EW Margolin R Francis J , et al. Evaluation of delirium in critically ill patients: Validation of the Confusion Assessment Method for the Intensive Care Unit (CAM-ICU). Crit Care Med. 2001;29(7):1370-1379. 10.1097/00003246-200107000-00012 11445689

[31] Ware J Jr. Kosinski M Keller SD . A 12-item short-form health survey: Construction of scales and preliminary tests of reliability and validity. Med Care. 1996;34(3):220-233. 10.1097/00005650-199603000-00003 8628042

[32] R: A language and environment for statistical computing. 2019. R Foundation for Statistical Computing, Vienna, Austria. Retrieved October from https://www.R-project.org/.

[33] Oh TK Song I-A . Trained intensivist coverage and survival outcomes in critically ill patients: A nationwide cohort study in South Korea. Ann Intensive Care. 2023;13(1):4. 10.1186/s13613-023-01100-5 36637567 PMC9839899

[34] Uehlinger DE Jakob SM Ferrari P , et al. Comparison of continuous and intermittent renal replacement therapy for acute renal failure. Nephrol Dial Transplant. 2005;20(8):1630-1637. 10.1093/ndt/gfh880 15886217

[35] De Corte W Dhondt A Vanholder R , et al. Long-term outcome in ICU patients with acute kidney injury treated with renal replacement therapy: A prospective cohort study. Crit Care. 2016;20(1):256. 10.1186/s13054-016-1409-z 27520553 PMC4983760

[36] Lins RL Elseviers MM Van der Niepen P , et al. Intermittent versus continuous renal replacement therapy for acute kidney injury patients admitted to the intensive care unit: Results of a randomized clinical trial. Nephrol Dial Transplant. 2009;24(2):512-518. 10.1093/ndt/gfn560 18854418

[37] Zhang L Yang J Eastwood GM Zhu G Tanaka A Bellomo R . Extended daily dialysis versus continuous renal replacement therapy for acute kidney injury: A meta-analysis. Am J Kidney Dis. 2015;66(2):322-330. 10.1053/j.ajkd.2015.02.328 25843704

[38] Kovacs B Sullivan KJ Hiremath S Patel RV . Effect of sustained low efficient dialysis versus continuous renal replacement therapy on renal recovery after acute kidney injury in the intensive care unit: A systematic review and meta-analysis. Nephrology (Carlton). 2017;22(5):343-353. 10.1111/nep.13009 28128881

[39] Zhao Y Chen Y . Effect of renal replacement therapy modalities on renal recovery and mortality for acute kidney injury: A PRISMA-compliant systematic review and meta-analysis. Semin Dial. 2020;33(2):127-132. 10.1111/sdi.12861 32149415

[40] Schefold Joerg C Haehling Stephan Pschowski Rene , etal. The effect of continuous versus intermittent renal replacement therapy on the outcome of critically ill patients with acute renal failure (CONVINT): a prospective randomized controlled trial. Critical Care. 2014;18(1):R11. 10.1186/cc13188 PMC4056033 24405734

[41] Liang KV Sileanu FE Clermont G , et al. Modality of RRT and recovery of kidney function after AKI in patients surviving to hospital discharge. Clin J Am Soc Nephrol. 2016;11(1):30-38. 10.2215/cjn.01290215 26681135 PMC4702218

[42] Soum E Timsit JF Ruckly S , et al. Predictive factors for severe long-term chronic kidney disease after acute kidney injury requiring renal replacement therapy in critically ill patients: An ancillary study of the ELVIS randomized controlled trial. Crit Care. 2022;26(1):367. 10.1186/s13054-022-04233-4 36447221 PMC9706988

[43] Gammelager H Christiansen CF Johansen MB Tønnesen E Jespersen B Sørensen HT . Five-year risk of end-stage renal disease among intensive care patients surviving dialysis-requiring acute kidney injury: A nationwide cohort study. Crit Care. 2013;17(4):R145. 10.1186/cc12824 PMC4055988 23876346

[44] Meersch M Küllmar M Schmidt C , et al. Long-term clinical outcomes after early initiation of RRT in critically ill patients with AKI. J Am Soc Nephrol. 2018;29(3):1011-1019. 10.1681/asn.2017060694 29196304 PMC5827600

[45] Paškevičius Ž Skarupskienė I Balčiuvienė V , et al. Mortality prediction in patients with severe acute kidney injury requiring renal replacement therapy. Medicina (Kaunas). 2021;57(10):1-9. 10.3390/medicina57101076 PMC8537734 34684113

[46] Vrettou CS Mantziou V Ilias I , et al. Quality of life, depression, and anxiety in survivors of critical illness from a Greek ICU. A prospective observational study. Healthcare (Basel). 2021;9(7):1-10. 10.3390/healthcare9070849 PMC8303596 34356227

[47] Combes A Costa MA Trouillet JL , et al. Morbidity, mortality, and quality-of-life outcomes of patients requiring > or=14 days of mechanical ventilation. Crit Care Med. 2003;31(5):1373-1381. 10.1097/01.Ccm.0000065188.87029.C3 12771605

[48] Euteneuer S Windisch W Suchi S Köhler D Jones PW Schönhofer B . Health-related quality of life in patients with chronic respiratory failure after long-term mechanical ventilation. Respir Med. 2006;100(3):477-486. 10.1016/j.rmed.2005.06.008 16039838

[49] Rimachi R Vincent JL Brimioulle S . Survival and quality of life after prolonged intensive care unit stay. Anaesth Intensive Care. 2007;35(1):62-67. 10.1177/0310057(0703500108 17323668

[50] Fukuhara S Lopes AA Bragg-Gresham JL , et al. Health-related quality of life among dialysis patients on three continents: The dialysis outcomes and practice patterns study. Kidney Int. 2003;64(5):1903-1910. 10.1046/j.1523-1755.2003.00289.x 14531826

[51] Villeneuve PM Clark EG Sikora L Sood MM Bagshaw SM . Health-related quality-of-life among survivors of acute kidney injury in the intensive care unit: A systematic review. Intensive Care Med. 2016;42(2):137-146. 10.1007/s00134-015-4151-0 26626062

[52] Mayer KP Ortiz-Soriano VM Kalantar A Lambert J Morris PE Neyra JA . Acute kidney injury contributes to worse physical and quality of life outcomes in survivors of critical illness. BMC Nephrol. 2022;23(1):137. 10.1186/s12882-022-02749-z 35392844 PMC8991933

[53] Oeyen S De Corte W Benoit D , et al. Long-term quality of life in critically ill patients with acute kidney injury treated with renal replacement therapy: A matched cohort study. Crit Care. 2015;19(1):289. 10.1186/s13054-015-1004-8 26250830 PMC4527359

[54] Salathé C Poli E Altarelli M Bianchi NA Schneider AG . Epidemiology and outcomes of elderly patients requiring renal replacement therapy in the intensive care unit: An observational study. BMC Nephrol. 2021;22(1):101. 10.1186/s12882-021-02302-4 33740897 PMC7980322

[55] Robinson CC Rosa RG Kochhann R , et al. Quality of life after intensive care unit: A multicenter cohort study protocol for assessment of long-term outcomes among intensive care survivors in Brazil. Rev Bras Ter Intensiva. 2018;30(4):405-413. 10.5935/0103-507x.20180063. (Qualidade de vida pós-unidades de terapia intensiva: protocolo de estudo de coorte multicêntrico para avaliação de desfechos em longo prazo em sobreviventes de internação em unidades de terapia intensiva brasileiras). 30652780 PMC6334490

[56] Falcão Antônio Luis Eiras Barros Alexandre Guimarães de Almeida Bezerra Angela Alcântara Magnani , etal. The prognostic accuracy evaluation of SAPS 3, SOFA and APACHE II scores for mortality prediction in the surgical ICU: an external validation study and decision-making analysis. Annals of Intensive Care. 2019;9(1). 10.1186/s13613-019-0488-9 PMC6353976 30701392

[57] Nunes RS Nicolini EA Menegueti MG Ferez MA Auxiliadora-Martins M Basile-Filho A . Comparison between saps 3 and APACHE ii in surgical patients admitted to a Brazilian ICU. Intensive Care Med Exp. 2015;3(1):A836. 10.1186/2197-425X-3-S1-A836

