# Supplementary material for: Long-Term Mortality and Health-Related Quality of Life After Continuous Versus Intermittent Renal Replacement Therapy in ICU Survivors: A Secondary Analysis of the Quality of Life After ICU Study
Source: J Intensive Care Med. 2024 Jan 9;39(7):636–45. doi: 10.1177/08850666231224392 (PMC11151712; doi:10.1177/08850666231224392)
Supplement: sj-docx-1-jic-10.1177_08850666231224392 - Supplemental material for Long-Term Mortality and Health-Related Quality of Life After Continuous Versus Intermittent Renal Replacement Therapy in ICU Survivors: A Secondary Analysis of the Quality of Life After ICU Study [file sj-docx-1-jic-10.1177_08850666231224392.docx]

**Electronic supplementary material**

**Long-term mortality and health-related quality of life after continuous versus intermittent renal replacement therapy in ICU survivors – are there differences?**

**Table of contents**

List of Participant sites (by state and hospital center)….……………………….……………………………. 01

Supplemental Table 1. Univariable analysis of factors associated with late mortality………... 02

**List of Participant sites (by state and hospital center)**

**Bahia**

*Hospital Geral Clériston Andrade* – Principal investigator: Lúcio Couto de Oliveira Júnior.

**Goiás**

*Hospital de Urgências de Goiânia* – Principal investigator: José Mario Meira Teles.

**Pará**

*Hospital Regional do Baixo Amazonas* – Principal investigator: Lívia Correa e Castro.

**Rio Grande do Sul**

*Hospital Ernesto Dornelles* – Principal investigator: André Sant’Ana Machado.

*Hospital de Clínicas de Porto Alegre* – Principal investigator: Silvia Regina Rios Vieira.

*Hospital Moinhos de Vento* – Principal investigator: Roselaine Pinheiro de Oliveira.

*Hospital Nossa Senhora da Conceição* – Principal investigator: Wagner Nedel.

*Hospital Santa Clara* – Principal investigator: Rodrigo Boldo.

*Pavilhão Pereira Filho* – Principal investigator: Daniella Cunha Birriel.

**São Paulo**

*Hospital do Coração* – Principal investigator: Alexandre Biasi Cavalcanti.

**Supplemental Table 1. Univariable analysis of factors associated with late mortality.**

|  | Mortality group  (no.=67) | Survival group  (no.=128) | Hazard ratio  (95% CI) | *P*-value |
| --- | --- | --- | --- | --- |
| Sociodemographic characteristics |  |  |  |  |
| Age (years) – median (IQR) | 71 (58-79) | 64.5 (48.8-74.2) | 1.02 (1.00 - 1.03) | 0.010 |
| Age >65 years – no./total no. (%) | 44/67 (65.7) | 64/128 (50) | 1.54 (1.01 - 2.34) | 0.043 |
| Female sex – no./total no. (%) | 29/67 (43.3) | 54/128 (42.2) | 1.03 (0.70 - 1.52) | 0.883 |
| Educational attainment (years) – median (IQR) | 8 (5-13.5) | 11 (5-11) | 1.00 (0.96 - 1.04) | 0.951 |
| Higher education^a^ – no./total no. (%) | 17/67 (25.4) | 27/127 (21.3) | 0.86 (0.56 - 1.33) | 0.507 |
| Monthly *per capita* household income^b^, USD – median (IQR) | 533.2 (262.6-1003.2) | 537.9 (347.7-1688.8) | 1.00 (1.00 - 1.00) | 0.212 |
| State of health before admission to the ICU |  |  |  |  |
| Charlson comorbidity index – median (IQR) | 3 (2-5) | 2 (0-3) | 1.13 (1.04 - 1.22) | 0.002 |
| High comorbidity^c^ – no./total no. (%) | 55/67 (82.1) | 71/128 (55.5) | 2.51 (1.45 - 4.36) | 0.001 |
| Previous dementia – no./total no. (%) | 5/67 (7.5) | 2/128 (1.6) | 2.17 (1.30 - 3.61) | 0.003 |
| Previous depression – no./total no. (%) | 17/67 (25.4) | 21/125 (16.8) | 1.38 (0.90 - 2.10) | 0.135 |
| Previous anxiety – no./total no. (%) | 14/67 (20.9) | 23/126 (18.3) | 1.11 (0.70 - 1.78) | 0.652 |
| Previous myocardial infarction – no./total no. (%) | 13/67 (19.4) | 15/128 (11.7) | 1.55 (8.47 - 2.84) | 0.150 |
| Previous congestive heart failure – no./total no. (%) | 13/67 (19.4) | 19/128 (14.8) | 1.33 (7.27 - 2.44) | 0.350 |
| Previous cerebrovascular disease – no./total no. (%) | 16/67 (23.9) | 10/128 (7.8) | 2.44 (1.39 - 4.28) | 0.200 |
| Previous diabetes with chronic complications – no./total no. (%) | 10/67 (14.9) | 11/128 (8.6) | 1.41 (7.21 - 2.76) | 0.310 |
| Previous mild chronic kidney disease ^d^ – no./total no. (%) | 6/67 (9) | 8/128 (6.2) | 1.29 (5.57 - 2.98) | 0.550 |
| Characteristics of acute critical illness |  |  |  |  |
| Risk of death at ICU admission^e^ – median (IQR) | 38.9 (19.9-72.8) | 33.1 (18.6-56.9) | 1.00 (1.00 - 1.01) | 0.286 |
| ICU Admission type: medical admission – no./total no. (%) | 50/67 (74.6) | 97/128 (75.8) | 0.99 (.075 - 1.30) | 0.927 |
| Sepsis or septic shock at ICU^f^ – no./total no. (%) | 34/67 (50.7) | 72/128 (56.2) | 0.87 (0.59 - 1.27) | 0.463 |
| ARDS^g^ at ICU – no./total no. (%) | 7/67 (10.4) | 17/128 (13.3) | 0.83 (0.43 - 1.60) | 0.581 |
| Organ dysfunction during ICU stay |  |  |  |  |
| Need of mechanical ventilation – no./total no. (%) | 40/67 (59.7) | 86/128 (67.2) | 0.81 (0.55 - 1.20) | 0.293 |
| Days of mechanical ventilation – no./total no. (%) | 3 (0-12) | 4 (0-10) | 1.00 (0.99 - 1.02) | 0.609 |
| Need of vasopressor – no./total no. (%) | 53/67 (79.1) | 102/128 (79.7) | 0.98 (0.61 - 1.57) | 0.923 |
| Need of parenteral nutrition – no./total no. (%) | 6/67 (9) | 11/128 (8.6) | 1.03 (0.52 - 2.02) | 0.932 |
| Need of blood or blood products transfusion – no./total no. (%) | 23/67 (34.3) | 46/128 (35.9) | 0.95 (0.63 - 1.44) | 0.824 |
| Delirium – no./total no. (%) | 28/67 (41.8) | 50/128 (39.1) | 1.08 (0.73 - 1.59) | 0.711 |
| ICU-acquired infection^h^ – no./total no. (%) | 20/67 (29.9) | 39/128 (30.5) | 0.98 (0.64 - 1.50) | 0.929 |
| ICU length of stay (days) – median (IQR) | 9 (6-19.5) | 10 (6-18.2) | 1.00 (0.99 - 1.02) | 0.412 |
| ARDS, acute respiratory distress syndrome; CI, confidence interval; ICU, intensive care unit; IQR, interquartile range (p25-p75); MMSE, Mini-Mental State Examination.  ^a^ Individuals holding a university degree.  ^b^ Using the purchasing power parity conversion (Brazilian real to U.S. dollar). Purchasing power parities are the rates of currency conversion that equalize the purchasing power of different currencies by eliminating the differences in price levels between countries https://data.oecd.org/conversion/purchasing-power-parities-ppp.htm.  ^c^ Charlson comorbidity index >2.  ^d^ Mild chronic kidney disease not hemodialysis dependent before ICU admission.  ^e^ The risk of death was calculated using established prediction equations for hospital death according to the Acute Physiology and Chronic Health Evaluation II score (APACHE-II) or the Simplified Acute Physiology Score-3 (SAPS 3).  ^f^ According to the sepsis-II criteria.  ^g^ According to Berlin criteria.  ^h^ Pneumonia, bloodstream infection, or urinary tract infection according to the European Centre for Disease Prevention and Control criteria. | | | | |
